# Acceptability and use of ready-to-use supplementary food compared to corn–soy blend as a targeted ration in an HIV program in rural Haiti: a qualitative study

**DOI:** 10.1186/s12981-016-0096-9

**Published:** 2016-02-17

**Authors:** Anne G. Beckett, Debbie Humphries, J. Gregory Jerome, Jessica E. Teng, Patrick Ulysse, Louise C. Ivers

**Affiliations:** Internal Medicine Residency, Department of Medicine, Brigham & Women’s Hospital, 75 Francis St., Boston, MA 02115 USA; Department of Medicine, Boston Children’s Hospital, Boston, MA USA; Partners In Health, Boston, MA USA; Department of Epidemiology of Microbial Disease, Yale School of Public Health, 60 College Street, Ste 318, New Haven, CT 06510 USA; Zanmi Lasante, St. Marc, Haiti; Division of Global Health Equity, Brigham & Women’s Hospital, 641 Huntington Avenue, Boston, MA 02115 USA; Department of Global Health and Social Medicine, Harvard Medical School, Boston, MA USA; Zanmi Lasante, 18A, Santo 18, Croix-des-Bouquets, Haiti

**Keywords:** HIV, Nutrition, Ready-to-use supplementary food

## Abstract

**Background:**

Ready-to-use supplementary food (RUSF) is increasingly used as a component of food rations for adults with HIV.

**Methods:**

We undertook a qualitative study to evaluate the acceptability and use of peanut-based RUSF compared to corn–soy blend (CSB) among adults living with HIV in rural Haiti who had been enrolled in a prospective, randomized trial comparing the impact of those rations. A total of 13 focus groups were conducted with 84 participants—42 selected from the RUSF arm of the study, and 42 from the CSB arm—using a guide with pre-designated core topics and open-ended questions.

**Results:**

We found that RUSF was highly acceptable in terms of taste, preparation, and packaging. Both types of food ration were widely shared inside and outside households, especially with children. However, while CSB was without exception stored with the communal household food supply, RUSF was frequently separated from the household food supply and was more often reserved for consumption by individuals with HIV.

**Conclusions:**

RUSF was a highly acceptable food ration that, compared to CSB, was more often reserved for use by the individual with HIV. Qualitative examination of the perceptions, use, and sharing of food rations is critical to understanding and improving the efficacy of food assistance for food-insecure people living with HIV.

## Background

### HIV and food insecurity

In both high- and low-resource settings, food insecurity and malnutrition are more prevalent among people with HIV/AIDS than their HIV-negative or untested counterparts [1]. The negative impact of food insecurity on the lives of HIV-infected individuals has been demonstrated at multiple levels, including access and adherence to treatment, antiretroviral medication (ARV) pharmacokinetics, clinical outcomes, and risk of transmission of HIV [2–9]. International multilateral and nongovernmental organizations, including the World Health Organization (WHO), now recommend the integration of nutritional interventions into comprehensive HIV treatment programs [10]; however, there are many gaps in the evidence available to guide this nutrition supplementation. One approach to nutritional supplementation is the provision of food rations, often including corn–soy blend (CSB), an inexpensive, blended mix that can be fortified with micronutrients [11]. Several large observational studies, including one in Haiti, have demonstrated that a food ration of CSB for adults with HIV is associated with improved food security and increased BMI [12], and also with improved adherence to HIV treatment, compared to no food ration [13, 14]. However, research has been more equivocal on the impact of food supplementation on CD4 count and overall survival of HIV patients [13, 15].

### Ready-to-use foods

Peanut-based ready-to-use therapeutic food (RUTF) was first used on a large scale in the 1990s as an alternative to the then-standard treatment for severe childhood malnutrition, F100 (a milk powder fortified with vitamins and minerals) [16]. RUTFs are nutritionally dense pastes, are generally individually packaged in aluminum wrapping, do not require potable water or cooking, can be stored without refrigeration, and have a higher calorie-to-weight and calorie-to-volume ratios than blended flours [17–19]. Ready-to-use supplementary food (RUSF) is similar to RUTF, but is intended to be a nutritional supplement given to vulnerable populations, or to prevent malnutrition. After randomized trials demonstrated that peanut-based RUTF was equally or more effective than F100 in promoting weight gain in malnourished children [20, 21], in 2007 the WHO and World Food Programme issued a joint statement advocating outpatient, community-based treatment of severely malnourished children with RUTF [22].

### Use of ready-to-use foods in HIV treatment programs

Given the success of RUTF in treating childhood malnutrition, interest has grown in the use of RUTF and RUSF in HIV treatment programs. Because it does not require preparation, it may be easier and more convenient for adults with HIV to consume; because it is individually packaged, it may dissuade sharing and lead to increased consumption by adults with HIV compared to CSB. Potential disadvantages of RUSF include unfamiliar or monotonous taste, cost (RUSF may be up to three times more expensive than CSF) [23], and lack of capacity for local production in many settings necessitating that the product be imported [16]. Data comparing the use of RUTF/RUSF to CSB or other macronutrient supplements in terms of their impact on key HIV-related outcomes are limited.

Our team recently conducted a randomized trial and found no difference in BMI, CD4 count, food insecurity, general health perceptions, quality of life, and household wealth index among people living with HIV receiving RUSF compared to those receiving CSB after 12 months [24]. We undertook a qualitative study embedded in that randomized trial to evaluate the acceptability and use of RUSF compared to CSB among people living with HIV in rural Haiti. We hypothesized that RUSF was acceptable, but that monotony might limit daily consumption of the prescribed ration. We also hypothesized that RUSF was more likely to be consumed by the target recipient (the adult with HIV infection) rather than their family members, when compared to CSB.

## Results

A total of 13 focus groups were held with 84 participants between November 2012 and May 2013. Forty-two of these participants were recipients of RUSF in the randomized study; the other half received CSB.

### Need for food rations

Many participants in both arms of the study expressed belief that food was an essential component of HIV care. They noted that ARVs increased their appetite, and many noted that the side effects of ARVs were more pronounced if taken in the absence of food.

I am taking a medication that makes me want to eat more.

I eat a lot because after I take the medication I am hungry.

After you swallow the pill you have numbness and it does not feel good, and your chest is hurting. So you should have something to eat after that.

Many participants noted that after the completion of the research study from which they received monthly food rations, they faced significant difficulty in acquiring adequate food.

Since the program stopped, we have not received any food…The food is what helps me out. My wife passed away and left me with the kids and I cannot work. It is basically me asking for handouts.

When the program was over, I was suffering a lot, because I did not have food.

### Acceptability of ready-to-use supplementary food

The RUSF was highly acceptable to the participants who received it based on favorable taste as well as a belief that it was helping them to have increased energy and weight gain. No participants who received it reported tiring of the taste or finding it monotonous after months of daily consumption. The Haitian Creole word for peanut butter, “manba,” was universally used to describe the RUSF.

It tastes sweet, it tastes like it has salt. We like it.

We did not get fed up with the manba. I would love to keep eating it!

One participant reported that at first he did not like the taste of the RUSF, but then grew to like it.

In the beginning, I really didn’t like it. Several days later, I liked it, I got used to it. I ate it every morning and before I went to bed, too.

Only two participants reported not liking the RUSF—one because it was too salty, and one because it made him feel nauseous.

The manba that you gave us in the program has too much salt. If they still want to give this manba, they should reduce the salt.

I feel light-headed when I eat it…. Actually, I like the taste of it. But when I eat it I feel light-headed and like I want to vomit.

Participants believed the RUSF was associated with weight gain.

When we started eating the manba, we gained weight…After one month, two months, we saw that we started gaining weight.

When one participant in the RUSF group was asked if the study were to be conducted again whether she would like to receive RUSF or CSB, she replied:

I would still like to be in the manba group because I want to build my body. Something that gives me more energy is better. The manba is something more special because it has a lot of vitamins.

Another participant replied to the same question with:

[Manba] has more vitamins, and if you eat two bags per day you feel full.

Many participants described one of the benefits of the RUSF as the strength and energy it imparted.

When I eat the manba, I feel strong.

When you eat manba it keeps you up for all day long. If you do not eat anything in the morning and you eat a bag of manba it keeps you up…’Kenbe ou.’ It is not energy exactly, it keeps you up!

### Acceptability of corn–soy blend

Almost all participants in the CSB group reported that the CSB distributed in this study was spoiled—some reported that it was spoiled upon receipt, and others reported that it spoiled quickly if not consumed.

At the beginning, the food I received was good. But after that the corn flour that I received was not good…It had bugs in it.

[The corn flour] had bugs, white little worms.

I used to eat it. When they gave us the corn flour, it did not have worms or bugs. If I kept it for a long time it would produce the worms and bugs. But if they gave it to me and we ate it very fast, it would not have time to grow worms and bugs.

Most participants consumed the CSB anyway; others considered it inedible.

If it has the little black bugs, we eat it. If it is little worms or maggots, we don’t eat it.

I gave it to pigs. I never ate it.

When participants in CSB groups were asked whether they would choose to receive RUSF or non-spoiled CSB in any future food assistance programs, many elected for non-spoiled CSB.

If the corn flour is good, I would choose corn flour.

Corn flour. It gives you energy. You can cook it with salt, sugar, and it gives more energy.

Because when you eat more corn flour you have more energy!

### Preparation of the food rations

Both RUSF and CSB were consumed in a variety of ways. The CSB was made into either soft corn porridge or hard corn meal, and was frequently mixed with vegetables. A favorite preparation was with meat, though this was often too expensive for participants to obtain. The RUSF was spread on bread or crackers, or mixed into other foods such as rice, beans, fruits, and vegetables. Most frequently, the RUSF was eaten straight from package with a spoon or squeezed directly from package to mouth. When food items in participants’ regular diet were available, the RUSF was used as a supplement to normal meals; frequently, however, participants reported that no food was available and RUSF was used as a substitute for morning and afternoon meals.

### Disclosure of illness associated with food rations

Participants in both the RUSF and CSB groups expressed concern that the receipt of food rations disclosed their status as having an illness, and that as a result, they were more vulnerable to stigma associated with illness, specifically HIV infection. One participant in the RUSF group noted that some persons enrolled in the program did not go to the depot to receive their monthly food ration for this reason.

Because in the neighborhood, when they get out of the car with a big sack of food, people in the neighborhood will say, “Oh, that person is sick.”

Many participants shared that they felt shame when picking up the food ration and employed strategies to conceal the activity.

Sometimes I get shamed and I hide. I pretend I am going to work the field. I put an empty bag on my back. I run around and go to get the food. When I come home, they point to me and say, “This is him, he went to get the food because he is sick.”

Me, I have a bike. I leave the bike somewhere far away from my house. So after I get the food, I put it on the bike and go to my house, so they think it is from the garden, it is not the food from the depot.

I would cover the sack they gave the food in from the depot. I would buy another empty sack to cover it so people do not know it is from the depot.

One woman in the CSB blend group reported that her husband did not know her HIV status. In order to minimize the risk of disclosing to him, she stored the food ration in her mother’s house.

My mom cooks it, and I go to her house and eat it.

### Sharing within the household

Not a single participant reported consuming the entire food ration (including the family ration) his or herself. One participant in the CSB blend group initially said that he lives alone and did not share with anyone, but then modified his statement to say that he sometimes shared with workers near his home. Most participants in both groups shared liberally with household members.

There are about 15 people in my house. Brothers, sisters, my mother and father, and my two children. Whatever I have, I share with them, and whatever they have, they share with me.

When I received the food…I always share the food with my family. The rest I take it for me and my children.

Everyone in the house eats more than me.

I eat a lot, but the kids eat more!

Eight people are living in my house. And four neighbors, sometimes I share my food with them. But the people who ate my food regularly are my kids.

All participants reported sharing some amount of the RUSF with their families. Sharing RUSF with children seemed to be so commonplace that it was often not even considered by participants as sharing at all.

No, we did not share it [manba] with anyone. We give it to our kids, but we don’t give it to everyone in the house.

I did not share it with other people. But I have a kid and I would give him some.

While participants always stored CSB with the communal household food supply, they frequently reported separating the RUSF from the household food supply in order to prevent rapid consumption by other household members.

I tuck it [manba] in my private room.

I put the manba in a bucket and I locked it and I would go away with the key. The rest of the food I put it where everyone can see.

With the manba, when I get it I normally hand everybody one, and then stash the rest inside.

I would hide the manba because otherwise people would eat all of it….. In a little closet, I locked it away. I would keep the key.

Many participants suggested that because they hid the RUSF they ultimately consumed a greater portion than they would have otherwise.

We hide it so we can eat more than the rest of the people in the house.

Who ate more? I ate more, because I am the one to give it out.

### Distribution outside the household

Many participants in both groups reported sharing the targeted ration items with neighbors. The most frequently cited reason for sharing with people outside the household was their common circumstances and either past or anticipated future dependence on these neighbors for food.

The reason that we share with the neighbors is because sometimes the neighbors don’t have money to buy food. Sometimes we don’t have money to buy food, and the neighbors share with us.

We are in the same situation. Sometimes when I do not have, they will share with me.

Other participants reported sharing food with less well-off non-household members.

I felt sorry for the neighbors who have nothing to cook at home.

Participants reported that there were often people waiting at the warehouse where the food was distributed, who asked for portions of the food ration.

When we went to the depot and got food… there were a lot of people standing around the depot asking for food. So we shared some with them. If the sack had not been open, there would not have been a way to share with them. But the sack was open, so we have to give something.

Other participants reported sharing food with non-household members because they feared ill will or retribution if they did not.

If you are making food at home, you have to share with the neighbors…It is a cultural thing, if you don’t share they would say you are cheap.

I have plenty of food at home, I have to share. Otherwise people would hate you, or would like to kill you.

Approximately one-third of the participants reported not sharing food outside their household. A few participants reported not sharing with neighbors because they felt they had an insufficient quantity of food to do so.

The food doesn’t even last for us inside the house!

The most commonly cited reason for not sharing the food ration outside the home was concern that the ration would be associated with illness, particularly HIV.

I didn’t share with neighbors because they said, “These people are sick.” Because we are receiving food every month from the hospital.

At the beginning, I shared with the neighbors. But then there were rumors to say I was sick, that I have an infection; that is why I am receiving this food. After I heard that, I kept the food in the house and did not share with anyone outside.

No, I do not share the food, because even if I do give the food, that person or someone else will say, “This person has HIV, that is their food.”

Other participants reported that they did not share the food outside the home because they believed people who knew they received the ration because they were sick would reject it.

I am not going to lie, I did not share. Other people say, “You have the disease, I’m not going to eat it.”

[I do not share] too often, because a lot of people are afraid of us.

A lot of times we do not give the food to the neighbors, because when we give to the neighbors they criticize us…They would say they don’t want to eat food from this person because of the illness.

Very few participants in either group reported exchanging or selling any portion of the food ration. A few participants said that they exchanged some of the rice and beans with higher quality rice and beans (in order to then mix the inferior and superior versions to make the end product more appetizing). A few participants reported selling some of the rice in order to buy a rice of better quality. However, almost all participants denied selling any of the food received, emphasizing that even with the addition of the food ration, they did not have sufficient food for their families.

I think that the patients would not sell the items because if you got food for a month, it’s not enough for a month for the family, so you would never sell it.

A few participants suggested that other participants might have needed to sell a small number of food items in order to afford transport home from the warehouse.

There are times that I saw with my own eyes, people who did not have money to go home from the depot, they would sell one or two cans so that they could have money to get home. Or to buy charcoal to cook the food.

## Discussion

In our study, RUSF was highly acceptable among people living with HIV in rural Haiti in terms of taste, preparation and packaging. Our study supports the findings of Ndekha et al. in Malawi, who found peanut-based RUSF “universally highly appreciated” among adults with HIV [25]. In contrast, Dibari and colleagues studied the use of RUTF among HIV-infected adults in Kenya, and while their data revealed positive perceptions of RUTF among participants—it was associated with increased strength, ability to return to work, and weight gain—participants frequently complained of taste, monotony, and gastrointestinal symptoms, and adherence to RUTF was low [26]. In Ethiopia, Olsen and team found that many adults with HIV who received peanut-based RUSF complained of nausea and vomiting that made them unable to take the full prescribed amount during the first few weeks, though most adapted to the taste [27]. Only one participant in our study reported nausea associated with RUSF, and very few participants described difficulty consuming the entirety of the prescribed food ration. One reason for this might be that that peanut butter is a staple in Haitian households, though the RUSF distributed in our study was reported to smell and taste noticeably different than the locally made peanut butter. In several of the previous studies of the use of RUTF/RUSF in adults with HIV, participants were initiated on ARV at the same time they were enrolled in the food supplementations study, and it is possible that some of the side effects attributed to the food product may have in fact been related to medication initiation. In our study, participants were initiated on ARV prior to enrollment in the study (median duration on ARV for participants receiving RUSF was 10.2 months), and so were unlikely to conflate side effects from ARV initiation with those of the RUSF [24].

We found that sharing of all food rations was typical, both within and outside of households, with a variety of reasons behind sharing. Our findings are consistent with those in Kenya, where more than half of the interviewed participants reported sharing RUTF with family and others [26], and in contrast to those in Ethiopia where most participants did not share RUSF with household members [27]. Participants in our study were most likely to share RUSF with children in their household: when some participants in our study spoke of “sharing” they understood it to mean when food rations were given to adults, as distributing some amount to children in the household was so prevalent that it was assumed to occur. The storage of RUSF within participants’ households was unique, and this was often related to attempts to minimize sharing. Unlike CSB, which was without exception stored with the communal household food supply, RUSF was frequently hidden or locked away by participants. It was also notable that the open sacks used to distribute CSB made some participants feel that they could not avoid sharing with people who waited and asked them for food at the warehouse. Studies in Niger and Ethiopia have suggested that RUSF may be more likely to be reserved for the target recipient and less likely to be shared with household members because it is regarded as having medicinal as well as food properties [27, 28]. The descriptions of RUSF shared by our participants did not reflect a perception of RUSF as medicine, but the behaviors described by participants suggest that RUSF was uniquely exempt in some ways from the social obligation to share food within the household. Understanding the interplay between social norms around food sharing and RUSF is important to understanding its use and efficacy in different settings.

Our study highlights concerns around disclosure of illness, particularly HIV infection, and the extent to which food assistance may be associated with this disclosure. These concerns have been noted in other studies: in the region of Kenya where Dibari and colleagues conducted their study, RUSF has become strongly associated with HIV, and many participants cited HIV-related stigma as a barrier to collecting and consuming the nutritional supplement [26]. In Ethiopia, the primary concern for participants receiving RUSF was the risk of their HIV status being revealed because of association with the nutritional product, leading participants to conceal the receipt of RUSF from other household members [27]. Understanding the risks of disclosure of illness can inform improved design of food assistance packaging and distribution in order to minimize vulnerability of the populations served.

Our group’s randomized food assistance trial hypothesized that RUSF might lead to improved nutritional and non-nutritional outcomes compared to CSB, but instead showed increased CD4 count, BMI, general health perceptions, and adherence to ARV among recipients of both food ration groups with no statistically significant difference in outcomes between the two groups [24]. Our qualitative data suggest that the lack of difference in outcomes between RUSF and CSB is not explained by a lack of acceptability or a higher likelihood of sharing of RUSF by the index patients. One factor that may have contributed to lack of difference in outcomes between RUSF and CSB is the extent to which the two food supplements displaced other food consumption. This qualitative study also highlights the importance of food assistance in general to this population of highly food insecure people living with HIV, through their repeated assertions that food assistance is an essential component to HIV treatment, and their reports of difficulty in obtaining adequate food for themselves and their families since the completion of the food assistance study.

We were surprised to note that a number of participants complained of CSB being spoiled upon receipt of the ration. The food rations were stored in approved food storage facilities that were regularly inspected, and food was distributed quickly upon arrival in the warehouse. Because this qualitative study occurred after food distribution ended, it was not possible to intervene with this issue for these participants, but a report was filed with the program managers to review the issue for any further food distribution programs.

There are some limitations to our study. The focus groups in this study were conducted 16–22 months after the randomized food assistance program had ended, and many participants had been previously enrolled in other food assistance programs, especially with CSB. Identifying the exact CSB food ration from the randomized trial was at times challenging. This was not the case for RUSF, which was not used in other food assistance programs for adults in the region.

## Conclusions

In our study of food insecure people living with HIV in rural Haiti, RUSF was a highly acceptable food ration. RUSF, like all food rations distributed in the study, was widely shared, but unlike CSB, it was frequently stored separately from the communal household food supply, and more often reserved for use by the individual with HIV in the household. Qualitative examination of the perceptions, use, and sharing of food rations is critical to understanding and improving the efficacy of food assistance for food-insecure people living with HIV.

## Methods

We conducted thirteen focus groups to explore the acceptability and use of RUSF compared to CSB among people living with HIV in a comprehensive HIV treatment program in rural Haiti. The study took place in the Artibonite Department of Haiti, a mostly rural area, 150 km from the capital Port-au-Prince, with an HIV prevalence of 2.3 % among adults aged 15–49 [29] and with high levels of food insecurity [30]. Participants were selected from persons enrolled in a larger prospective, randomized trial in which adults with HIV on ARV were randomized to receive either a peanut-based RUSF or a CSB ration for 12 months. Individuals were eligible for the randomized control study if they were documented to have HIV infection by standard laboratory procedures, lived in the geographic catchment area of services provided by non-governmental organization Partners In Health, were ≥18 years of age, and had started ART in the 24 months prior to study enrollment. Individuals were excluded if another household member was also eligible for food assistance or if they were pregnant at the time of enrollment. Participants in the “CSB” arm received a monthly food ration comprised of a bulk 30-day supply of 200 g CSB, 20 g oil and 15 g sugar per day. Participants in the “RUSF” arm received a 30-day supply (60 sachets) of peanut-based RUSF. Education included the instruction that these components were for consumption by the individual with HIV infection. All participants also received a “family ration” comprised of a bulk 30-day supply of 300 g rice, 50 g beans, 25 g oil, and 5 g salt per person per day for a family of five, to be shared with other persons as desired, to offset the nutritional needs of the family. The two ration types were equal in number of calories. Medical care and comprehensive HIV treatment were provided by Partners In Health in collaboration with the Haitian Ministry of Health, free of charge to patients.

### Sampling and recruitment

For the first eight focus groups, we used stratified random sampling to select an approximately equal number of participants from each arm of the primary study. Nurse coordinators contacted the selected participants to invite them to participate in focus groups, and informed consent was obtained. To further explore some of the themes that emerged in these initial groups, five additional focus groups were held with a combination of persons who had already participated in focus groups and new participants randomly selected from the two study arms.

### Data collection

Each focus group was comprised of five to twelve participants from a single arm of the study; participants from the two arms were not mixed. We developed a semi-structured focus group guide with pre-designated core topics including household food practices, perception and acceptability of food ration items, and preparation and use of food ration items. We adapted the guide throughout the course of the focus group sessions until saturation of themes was achieved. Focus groups were conducted in English and Haitian Creole by one of the authors (AGB), with the assistance of a Haitian Creole translator. The focus group discussions were digitally recorded and we produced complete transcripts in English, which were entered into the qualitative data software HyperRESEARCH (ResearchWare, Inc., Randolph, MA).

### Data analysis

We analyzed the data using an inductive approach informed by grounded theory described by Ware and colleagues, through which we reviewed the transcripts multiple times to both obtain a sense of the whole and to develop a list of categories of responses [31]. We tagged the data for these categories of responses, and then grouped participants’ statements by category and re-reviewed. We compared and sorted the categories and then condensed them into broader themes. We actively searched for deviant cases. Observation notes recorded by the focus group moderator and the study coordinator were reviewed and incorporated into the analysis.

### Ethics, consent, and permissions

This study received approval from the Yale University Human Investigation Committee (New Haven, CT), Partners HealthCare Institutional Review Board (Boston, MA), and the Zanmi Lasante Institutional Review Board (Cange, Haiti). All study participants gave verbal confirmation of their informed consent prior to focus groups.
